# Some Things I Have Learned About Aging by Studying the Life Course

**DOI:** 10.1093/geroni/igx014

**Published:** 2017-09-27

**Authors:** Richard A Settersten

**Affiliations:** Human Development and Family Sciences, Oregon State University

**Keywords:** Age, Cohort, Diversity, Gender, History, Inequality, Interdependence, Interpersonal, Relationships, Subjective Aging

## Abstract

“Aging” and the “life course” are distinct but complementary phenomena that inform one another. Building on this insight, this essay conveys some lessons the author has learned about aging by studying the life course. These include that (1) age is a salient dimension of individual identity and social organization; (2) a reconfigured life course brings reconfigured aging; (3) old age is a highly precarious phase of life; (4) difference and inequality are not the same, but both can accumulate over time; (5) aging is gendered; (6) aging is interpersonal, and “independence” is an illusion; (7) “choice” and “responsibility” can be dirty words; (8) much of aging is in the mind—it is imagined and anticipated; and (9) history leaves its footprints on aging, and the future of aging is already here. These lessons culminate in a final insight: that to understand personal aging, gerontologists must look *beyond* the personal, for much of the relevant action is to be found in social experience.

**Translational Significance**

Social forces, from social relationships to institutions and policies, demography, and historical change, shape individual and population aging. The strong emphasis on personal factors in gerontology can lead researchers and practitioners to overlook the fact that these “personal” factors are often interpersonal in nature and have social determinants and consequences.

Gerontology has been central to the development of a life course perspective because the subject matter demanded it. Old age^1^ as a life phase, is inherently different from earlier phases because there is a long past that must be taken into account. That long past reflects the fact that aging is a lifelong process. What has been called the “personological” paradigm of life course research (Dannefer & Settersten, 2010) attempts to account for these temporal connections. For example, there is a great deal of current interest in linking childhood socioeconomic status and family characteristics to later-life health, explorations that are made possible by the availability of long-ranging longitudinal data and by gathering retrospective life history data.

But a life course perspective on aging is much more than understanding the shadow of the past or studying a phenomenon “over the life course,” a phrase that can be found alongside just about any topic today. What has been called an “institutional” paradigm of life course research (Dannefer & Settersten, 2010) instead emphasizes the role of *social* forces in structuring the life course—related to spheres of family, education, and work, and related to social policies and social change, that open and close opportunities and pathways through life. The dominant focus on individuals in aging research, and on age-progressive decline in function, often leads scholars and practitioners to lose sight of these powerful social aspects of aging.

“Aging” and the “life course” are therefore different but complementary. Building on this insight, I share just a few lessons I have learned about aging by studying the life course. The essay is organized so that more foundational lessons come first, and that content flows naturally from one lesson to the next.

## Age is a Salient Dimension of Individual Identity and Social Organization

Gerontologists have an uneasy relationship with age. On one hand, age lies at the heart of scholarship on aging. It is such a taken-for-granted part of our scientific lenses that we fail to notice or question its presence as it sits comfortably in our models. On the other hand, some of the most dominant rhetoric of the field sends quite a different signal: that age is not something to be embraced as much as something to be disregarded or even transcended. Culturally, societies like the United States also promote an ideology that age is—or should be—irrelevant to personal ventures and outcomes.

### Age as a Property of Individuals

There are two primary approaches to age in aging research. The first and dominant approach takes age to be a property of individuals or as a means for classifying people into groups. Here, age is often not well theorized. When it is, it is generally assumed to bring decline or risk in various domains (such as cognitive or physical health), but it can also be connected to possibilities of growth (such as wisdom) or considered a buffer of risk (such as having accumulated experience that brings a wider repertoire of coping strategies).

Investigators must think more critically about why and how age matters for the phenomena being studied—and to explain the connection between age and aging. Chronological age is naturally enmeshed with aging because aging seems to be about advancing *in* age. Researchers often use age as a proxy for things that might be age-related but have not been measured. For example, it might be a placeholder for an individual’s psychological maturity, social roles and responsibilities, or health status. But age *itself* is not necessarily the issue. Age is simply convenient (everyone has one), practical (it is easily measured), and portable (it is always with us, though its appearance can be manipulated; for classic discussions of age, see Boyd & Dowd, 1988; Neugarten & Hagestad, 1976). Age is also subjectively experienced. Indeed, a body of research on “age identification” compares individual’s perceptions of how old they look, act, feel, or desire to be relative to how old they actually are. Subjective ages have been linked to health, personality attributes, and behavioral dispositions, and they shape the goals people set and pursue (for illustrations, see Diehl & Wahl, 2015).

### Age as a Property of Social Organization and Social Life

The second approach takes age to be a property of social organization and social life. The focus on individuals in aging research leads gerontologists to overlook or have at the periphery social phenomena that have direct and indirect bearings on aging processes and outcomes (see Settersten & Godlewski, 2016). These include:

- *Age and life phases:* members of a culture use age to divide life into distinct phases. Individuals are aware of their and others’ movement into and out of these phases, and identities, relationships, and expectations are based on these phases.
- *Age timetables for life transitions:* members of a culture are subject to social scripts of life that include expectations for the timing and sequencing of major transitions.
- *Age as a right and benefit:* ages are embedded in laws and policies related to education, work, family, and health. With respect to aging, discussions of legal ages often center on questions of how late in life these rights and obligations should be maintained, or the conditions under which they should be revoked.
- *Age and the allocation of social roles and activities:* age is a basis for organizing education, work, retirement, and leisure roles and activities, with institutions and markets catering to different age groups.
- *Age segregation and integration:* age-based roles and activities bring the possibility that, outside the family, age groups are largely separated from one another, which raises concerns about the costs of age segregation and the benefits of age integration for individuals and societies.
- *Ageism and age stereotypes:* age is a basis for formal or informal discrimination, or for negative or positive stereotypes. (Note: Younger people experience these things too!)
- *Age conflict and cooperation*: there may be conflict among age groups when there are limited public resources or “generation gaps” in values and attitudes, or cooperation among age groups when they have a common stake in social and political issues.

These points make it clear that age is a property of social systems, not just individuals. Gerontology needs dedicated inquiry into both aspects of age, which affect but are distinct from aging.

## A Reconfigured Life Course Brings Reconfigured Aging

The last century brought revolutionary reductions in mortality, morbidity, and fertility. These demographic changes created aging societies and transformed the aging experiences of individuals and families. They gave rise to the very concept of the life course and continue to permit its reorganization. The later years are not unique in this regard. Every period of life is being rewritten—what it means to be a child, adolescent, young adult, and middle aged too.

The changes occurring in other life periods have implications for aging. Consider the “tripartite” (Kohli, 2007), or three-box, structure of the life course with education in early life, work in the middle, and retirement at the end. The boundaries of these boxes have been altered and are even eroding, but it persists as a model for organizing lives and expectations. For example, the front end of adult life has seen major extensions in education and later entry into full-time work (Settersten & Ray, 2010), which in turn mean that recent cohorts of young adults are getting a slower start to savings and pension-building, potentially reducing resources in later life or delaying retirement. These risks are exacerbated by the fact that gains in longevity have also lengthened the retirement period. The extended support that parents now provide to their young adults for higher education and training, housing, and other purposes can strain family resources and constrain parents’ options and choices regarding work and retirement.

In the middle of life, technological and labor market changes have made it necessary for many working adults to return to school or seek training to remain competitive. This need has especially grown as “lifetime” models of work—in which employers and employees invest in a long-term partnership, and employee longevity translates to better wages, job security and mobility, and pensions—have vanished (Hardy, 2011). Stable work has itself become uncertain, and many jobs no longer carry health insurance or benefits, or the levels of protection they once did. This shift coincides with “contingent” models of work, which are characterized by time-bound contracts with no promise of employment beyond those parameters—or no contracts at all (Kalleberg, 2011). These changes, too, are likely reducing resources in later life and delaying retirement.

In later life, many older people are unaware of when and how to transition to retirement. A more gradual or prolonged transition to retirement is not always a choice as much as an immediate economic necessity or a reflection of worry about how much life will remain at the end of the money. Gauging the end of life is difficult, as people do not know whether they will live 5, 15, or 25 years. The “Great Recession” also took significant tolls on the savings and assets of many working-aged or already-retired people (Van Horn, Corre, & Heidkamp 2011), disrupting or undermining retirement plans and options.

Demographic change has in many countries also transformed social relationships. The duration of family relationships today is particularly striking. However, there are many countries in which life expectancy is under age 80, especially for men (National Institute on Aging, 2011), and in which there are serious concerns about the future longevity and health of cohorts of children and young adults, such that the gains that have been enjoyed by older cohorts may plateau or reverse. In some regions of the world, and even for subpopulations in seemingly well-positioned nations like the United States, a long life cannot be counted on—whether due to poor health conditions, high rates of poverty, high rates of infant and child mortality, violence, or political and economic upheaval.

## Old Age is a Highly Precarious Phase of Life

Being able to count on long life is not the same as being able to count on how the later years will be experienced. Old age is embodied with so much possibility, yet its potentials, if they are to be realized, depend on some Big Ifs that cannot be predicted or controlled. These contingencies relate to life, health, and resources: *if* we are still alive, healthy, can manage financially, can live independently, have support from children, and so on. As these contingencies come undone, so do the futures that have been counted on.

Old age spans multiple decades, and is often comprised of earlier and later phases that are extremely different. This is reflected in what historian Laslett (1989) first called the “third” and “fourth” ages of life, terms which have since infiltrated gerontology. These “ages” were produced by demographic conditions noted earlier. The third age can last several decades and is viewed as a time of opportunity and activity. It is a period during which most people no longer have childcare or work responsibilities, but are in good health. The fourth age, in contrast, involves major encounters with illnesses and is, as an ideal for many people, often followed in short order by death (the so-called “compression of morbidity,” see Fries, 2005). Trends toward medicalization (that is, life-extending treatments and life-saving interventions) are lengthening the fourth age. The transition from the third to fourth age is difficult, but not necessarily permanent. Individuals in the fourth age can move back into the third, for example, if debilitating conditions are cured or go into remission. Attempts to increase "healthspan" over "lifespan" are essentially about how to extend time in the third age.

These categories underscore important distinctions among older age groups. Because people of a particular age are so variable in health and other statuses, it is problematic for these “ages” to be defined in chronological terms, as they sometimes are. The same is true of the terms “young-old” and “old-old,” which have been fixtures in the aging literature since Neugarten (1974) first coined them. These labels were actually intended to separate elders who are relatively healthy, affluent, and active from those who are not—*irrespective* of their ages.

## Difference and Inequality Are Not the Same, but Both Can Accumulate Over Time

The greater heterogeneity of older people on many types of outcomes (e.g., health, income, assets), relative to younger groups, raises an important question: does it reflect individual differences or social inequalities (for discussion, see Dannefer & Settersten, 2010)? Both may develop as a time-based process. Where *individual differences* are concerned, the life experiences of members of a birth cohort “fan out” over time, culminating in profiles that are arguably as unique as their fingerprints. But *inequality* also tends to increase with age due to social stratification and exclusion, and to the accumulation of advantage and disadvantage (Dannefer, 2003; for illustrations related to racial and ethnic health inequalities in later life, see Ferraro, Kemp, & Williams, 2017). Much of the important action, however, seems less about cumulative disadvantage (i.e., the poor getting poorer) than it is about cumulative advantage (i.e., the rich getting richer), and the position of the disadvantaged *relative* to the advantaged. 

Social class, race, and gender are central to these processes, which begin in early family and learning experiences, including preschool and primary and secondary schools, and continues as individuals move through higher education, work organizations, health care institutions, and housing markets. These settings generate inequality because they are often structured to regulate access to scarce and desirable opportunities and resources based on these social categories, but these processes operate in subtle and hard-to-detect ways (Dannefer & Settersten, 2010). The stark and growing inequalities among children and youth in the United States, as well as in the working-aged adult population, mean that the inequalities among older people are only likely to increase in the future as these cohorts age.

## Aging is Gendered

One key dimension of difference and inequality in an “intersectionality” approach is gender. I single it out because gerontologists often generate models and treat processes of aging as if they are unisex. But there are aging *men* and aging *women* (and gender identities that are more complex), and life course experiences are heavily gendered. Men and women attach different social meanings to age and aging, use different guidelines to measure the progress of their lives, and are subject to different expectations and evaluations (Settersten & Hagestad, 2015). There are also strong and persistent cultural differences in the valuation of aging bodies, including the “double standard” of aging in which the physical signs of aging often accentuate a man’s social capital but for women take it away (Bell, 1970; Sontag, 1972).

One cannot help but wonder whether women’s lifelong experiences in more diverse and multiple roles might make it easier for women to adjust to change and manage the hardships and uncertainties of aging. It is common to hear about the tolls that women’s earlier investments in family life, whether forced or chosen, take on their careers and economic well-being. But these investments also seem to bring social dividends in later life, as women are more likely than men to be embedded in larger and supportive networks. The social well-being of older cohorts of men has been dependent on having wives who long maintained family relationships. My excitement about the emergence of the “new father” in younger cohorts is not only that greater investments in fathering might improve the well-being of children. It is that these investments might offer a gateway to men’s development—and eventually produce new kinds of older men: men who are more socially integrated and have deeper connections to children, family, and community. More fluid views of gender and gender identity among young people today may similarly transform the future aging experiences of both men and women.

Although the gap in the length of men’s and women’s lives has begun to close, mortality at every age is higher for males and the sex ratio naturally declines with age (Howden & Meyer, 2011). To be very old, then, is more often a female experience, and to be a long-lived society is to have a “feminized” aged population. I have always loved the idea, first put forward by Rossi (1986), that societies with growing populations of older women might be more humane, tolerant, and compassionate. Some of the most interesting questions about an aging society are not only about what it means for older people, but what it means for children and youth (see also Uhlenberg, 2009).

The long-standing sex differential in mortality also affects individual aging through the composition of social networks and the presence of a spouse. For example, it has been more typical for women than men to experience widowhood. This is not only the result of the fact that men die earlier, but also that women tend to marry older men. The different morbidities of men and women also mean that “men die quicker, women get sicker,” as the saying goes. That is, men more often die with little or no warning, whereas women more often live for many years with chronic illnesses (Hagestad & Settersten, 2016). The naturally declining sex ratio, which shifts significantly in the 70s and sharply in the 80s, means that the social worlds of older people, as well as care institutions, are heavily populated with women. Rather than create theories or conduct research that ignores or “controls” for gender, these examples all point to the need to place gender in the spotlight and rigorously account for it.

## Aging is Interpersonal, and “Independence” is an Illusion

Much of “personal” aging is actually *interpersonal* (Hagestad & Settersten, 2016). Our lives are rarely stories of “I” or “me,” but of “us” and “we.” Boldly, one could even argue that there is no such thing as an “individual” life. From birth to death, our lives are enmeshed with others and inherently social. The many decades of adult life are heavily shaped by relationships in which our own welfare is inextricably dependent on the choices, behaviors, and resources of others, and in which the welfare of others is inextricably dependent on ours (e.g., Elder, Shanahan, & Jennings, 2015; Settersten, 2015). These interdependencies can also be reinforced in or undermined by social policies (for illustrations, see Hagestad & Dykstra, 2016). Relationships significantly constrain our lives, but they also bring life much of its meaning. The later years are no exception.

The reality of interdependence as a hallmark of adult life stands in direct contrast to one of the most cherished cultural values in the United States: independence. Aging enters this dynamic because it threatens independence. There is a mistaken assumption that once people have reached adulthood they are completely independent until old age, when failing health may make it necessary to depend on others. The need to rely on care from others is even sometimes described as “burden” because it violates this cultural value, which is underscored by the high premium placed by the government and public on personal responsibility and self-reliance, to which I now turn.

## “Choice” and “Responsibility” Can be Dirty Words

In many respects, aging and old age in “liberal market states” such as the United States are “private troubles” more than “public issues,” to use C. Wright Mills’ (1959) famous phrases. That is, beyond basic protections provided by old-age policies, the later years are to be managed using whatever resources one has or can marshal for their own well-being. This is especially true of the vast space between childhood and old age, when there are limited and temporary supports. The neoliberal emphasis on personal responsibility seems only likely to grow as public resources dwindle and are in question economically and politically.

Gerontologists and aging advocacy organizations have been prophets of “productive,” “active,” and “successful” aging. This emphasis on positive aging reinforces the notion that individuals are responsible for their aging outcomes—exemplified, for example, by current talk of the need to “disrupt aging” through personal choices, promoted by the CEO of AARP (Jenkins, 2016), and by the commercial “anti-aging” industry (Flatt, Settersten, Ponsaran, & Fishman, 2013). Yet, many aspects of aging are not within individuals’ control and are, as a life course perspective reveals, rooted in social forces far outside of them.

People *do* make choices and engage in behavior that can help or hurt them. The danger of overly deterministic sociological perspectives, including theories of cumulative advantage and disadvantage, is that they allow little place for individuals’ “agency,” or for “off-diagonal” outcomes or outliers. But these messages about positive aging, which so exclusively emphasize choice, in contrast, can be damning because they communicate that *defying* aging, through one’s own efforts, is the primary way to *do* aging. There are some things about aging that cannot be escaped or postponed. These messages do a disservice to older people because they deny the very real and dark sides of aging—not only normative physical and cognitive declines that are difficult for most people, but also serious conditions that are debilitating or disabling for many people. These dark sides must be acknowledged if individuals and families are to prepare for and better manage them.

## Much of Aging Is in the Mind—It Is Imagined and Anticipated

Aging is a lifelong process, and old age is something we spend most of our lives moving toward. Much of the relevant action is therefore to be found in the mind—it is imagined and anticipated. No matter how young or old we are, the shadow of the future is always on the horizon. Aging brings a shorter time horizon and, with it, a premium on time left (see also Carstensen, 2006). Much of our behavior today is oriented toward things we would like to happen, or that might happen, in the future. In the Western world, planning is central to every period of life. Where aging is concerned, marriage and family planning (and divorce and remarriage too) may be done with an eye toward later resources or care needs; retirement and health care planning are salient during work life; estate, health care directives, and funeral planning are pressing concerns of the retirement years. So many of life’s transitions involve “anticipatory socialization,” which provides a sense of what might lie ahead and an opportunity to get ready. In the second half of life, these changes might include menopause for women, becoming grandparents or great-grandparents, retiring, providing care to others, encountering chronic and serious illnesses, needing care from others, being widowed, or preparing to die.

The failing health and death of parents and loved ones, and even ourselves, are also good examples of the power of anticipation. Historical declines in morbidity and mortality have made major illnesses and death more predictable in that they are now largely confined to later life. But we cannot know exactly how long we and others will live or have good health, and we probably overestimate it. Still, our ages may prompt us to think in more conscious ways about time left, and to change our behavior now by visiting or phoning more often. Couples wonder about which person will pass first and what it will mean for the survivor; children wonder the same about their parents. These are just a few of many examples of the fact that much of our subject matter in gerontology demands a stronger science of anticipation at the level of individuals and relationships.

## History Leaves Its Footprints on Aging, and the Future of Aging Is Already Here

The field of aging is relatively young. Most knowledge of human aging has been based on cohorts born in the first half of the 20th century—a century punctuated by major historical events and social change related to war, economic hardship and prosperity, health epidemics, civil rights reforms, and innovations in medicine, communication, and transportation. These events and changes may therefore be “hidden variables” (Spiro, Settersten, & Aldwin, 2016) underneath the knowledge base. We do not know how much this knowledge will apply to future cohorts whose historical experiences have been very different.

One way to increase our understanding is to become more intimately acquainted with younger cohorts—from younger Boomers who are perched on their later years and rather distinct from older Boomers, down to “Millennials” on the front end of adulthood. For example, Millennials are experiencing prolonged transitions to adulthood, with later patterns of school completion, labor force participation, partnering, and parenthood (e.g., Settersten & Ray, 2010). Their life aspirations and worldviews on a wide range of social and political issues also differ significantly from previous cohorts. One can—and should—be skeptical of the value and validity of broad generational labels because they coalesce a very heterogeneous group of people under a single banner. But there is also no question that successive cohorts will challenge much of what we understand about aging, and there is much to be gained in building a more “anticipatory gerontology” (Cain, 2003) by projecting the future of aging at the societal level too. After all, the nonagenarians of 2106 have already been born (Dannefer & Settersten, 2010).

## To Understand Personal Aging, Geronto logists Must Look Beyond the Personal

In conclusion, “aging” and the “life course” are distinct but complementary phenomena that inform one another. I have shared just a few lessons I have learned about aging by studying the life course. Chief among these insights is that both individual and population aging are heavily conditioned by social factors. The strong focus in the literature on personal factors associated with individual aging brings the risk of losing sight of the social. The need to keep social factors in focus is heightened by reductionist tendencies in science and the rush toward genomes and genetics. A tendency in disciplines such as psychology and biology is to dismiss external forces as being too unwieldy to measure or to assume that they are already reflected in lower-order phenomena. Social gerontologists must make the social world visible, filling in the “black box of the actor” (Mayer, 2004) by unearthing individuals’ awareness of the social constraints and incentives that affect their actions, as well as the “black box of structure” (Settersten, 2009) by generating more precise accounts of how a wide range of social settings shape and set parameters on human aging. To understand personal aging, gerontologists must look *beyond* the personal.

## Conflict of Interest

None reported.

## Footnotes

1 My use of the term “old age” here is intentional. To refer to a person—even to oneself—as “old” in the United States today is culturally taboo. In fact, the American Psychological Association’s guidelines for psychological practice, as well as the style guidelines of their publication manual, recommend the term “older adults,” which is also used by most gerontologists. The use of the term “old age” in describing a life period also seems to have become taboo among gerontologists, who instead use softer terms such as “later life” or “older age.” The use of such terms is consistent with the reality that age is a relative concept—individuals become older with the passing of time, and are older or younger with respect to others. This language is peculiar, though, because it contributes to the denial of aging and finitude. Human beings do become *old*, and *old age* is, in most societies, recognized as distinct period of life. In some societies, however, “old” and “old age” are not only embraced by individuals, but also bring social stature. I have adopted the new convention to describe people, but I have in some instances deliberately used the term “old age” for the life period. The avoidance of age-based language is, in the context of developmental and life course studies, rather unique to gerontology. It is well-intentioned in that it seems to be done out of respect—precisely because the term “old” is interpreted as devaluing and demeaning. But this practice reflects an “anti-aging” culture which, in trying to combat ageism, produces a new brand of it (see also Andrews, 1999). It is ironic that a field so entangled with age so firmly resists language related to it. Gerontology has uncomfortable relationships with both age and aging, and our field would benefit from more rigorous dialogue about its sources and solutions.
